# Infected thoracoabdominal aortic aneurysm related to an implanted long-term arterial catheter for chemotherapy: a case report

**DOI:** 10.1186/s13256-021-02661-4

**Published:** 2021-02-21

**Authors:** Kiyoshi Takemoto, Michitaka Nakamura, Masaaki Sakuraya, Tomonori Yamamoto, Wataru Iwanaga, Kazuaki Atagi, Kazuo Yamanaka, Takeshi Matsuyama

**Affiliations:** 1 Division of Critical Care Medicine, Nara Prefecture General Medical Center, Shichijyonishi 2-897-5, Nara City, Nara Japan; 2 Department of Critical Care Medicine, JA Hiroshima General Hospital, Hiroshima, Japan; 3 Department of Cardiovascular Surgery, Nara Prefecture General Medical Center, Nara City, Nara Japan

**Keywords:** Infected thoracoabdominal aneurysm, Implants, Arterial catheter

## Abstract

**Background:**

An infected aortic aneurysm is a rare and life-threatening vascular condition with a high incidence of arterial rupture and recurrence even after treatment. One of the most common causes of an infected aortic aneurysm is catheter-related bloodstream infection. Although infection due to indwelling catheters is possible, the incidence of this is rare, especially for long-term implanted arterial catheters.

**Case presentation:**

A 78-year-old Japanese man with a past medical history of rectal cancer with metastasis to the liver presented to our hospital as a result of low back pain. Remission had been achieved following surgery and adjuvant chemotherapy via an implanted catheter for arterial infusion. However, the original catheter that was inserted from the femoral artery to the hepatic artery via the celiac artery was still present more than 10 years after diagnosis, without being replaced, in case of a recurrence. On the day of admission, computed tomography scan of the chest and abdomen with contrast revealed an irregularly shaped aortic aneurysm at the origin of the celiac artery and a partially expanded common hepatic artery with disproportionate fat stranding along the implanted arterial catheter without extravasation. Although the initial impression was an impending rupture of the acute thoracoabdominal aortic aneurysm, a catheter-related infection was considered as a differential diagnosis. Surgery was performed, which revealed a catheter-related infected aortic aneurysm based on images along the catheter, pus cultures, and tissue pathology examination results.

**Conclusions:**

This is an extremely rare case of an infectious aneurysm caused by prolonged implantation of an arterial catheter for chemotherapy. It should be noted that an indwelling arterial catheter not only causes bloodstream infections but can also cause an infection of a thoracoabdominal aortic aneurysm.

## Background

An infected aortic aneurysm a rare life-threatening vascular condition which can eventually result in rupture of the arterial wall if left untreated. When infected aortic aneurysm is suspected, immediate confirmatory diagnosis and definitive treatment are essential [1]. As there are no definitive diagnostic criteria for infected aneurysms, radiographic imaging, blood and tissue cultures, and tissue pathology examinations must be evaluated. Negative blood cultures are not enough to exclude infected aneurysm because positive blood cultures can be obtained in only 50–70% of patients with an infected aneurysm [2, 3]. Despite report of venous and arterial infections aneurysms due to catheters [4], we could not find published works reporting infected aneurysms due to long-term implanted catheters. This was an extremely rare case of an infectious aneurysm caused by prolonged implantation of an arterial catheter. This case report suggests that an indwelling arterial catheter not only causes bloodstream infections but can also cause an infection of a thoracoabdominal aortic aneurysm.

## Case presentation

A 78-year-old Japanese male presented to the emergency department of our hospital with low back pain on exertion for 1 week. The pain was described as dull and gradually worsens. Although the location was near the thoracolumbar spine, he denied radiation of the pain to any parts of the body. Severity of pain using a numerical rating scale was 10/10 at the day of admission. The character and intensity of the pain were not affected by changes in physical movement or by rest. He denied any other symptoms such as fever, nausea, dysuria, hematuria, abdominal pain, and leg numbness during his clinical course.

He had a past medical history of rectal cancer with liver metastasis and had undergone surgery and chemotherapy. At the time of diagnosis, rectal cancer was stage IV (TNM classification of malignant tumors; T3N2M1), grade 3, and was revealed to be adenocarcinoma during histopathology. Liver metastasis affected segments 3 and 6. He underwent low anterior resection of the rectum and resection of the affected liver segments. He then underwent chemotherapy using fluorouracil that was arterially infused through a catheter inserted into the femoral artery and implanted into the hepatic artery through the celiac artery. He initially had good response to treatment but 2 years after diagnosis, he had a recurrence of liver metastasis. He underwent partial resection of segment 6 of the liver and was followed by chemotherapy using FOLFOX6 + bevacizumab protocol instead of arterial infusion. After finishing chemotherapy, he achieved complete remission 11 years after initial diagnosis. As a result of the possibility of another recurrence, the catheter remained in place without being replaced. His other past medical history was hypertension and he remained on amlodipine 5 mg daily and imidapril 5 mg daily. Social history revealed that he had smoked approximately 10 cigarettes a day for 50 years and drank alcohol occasionally. Family and environmental history was unremarkable. His employment history was an office worker, but he retired at the age of 60 and has not worked since then.

**Fig. 1:**
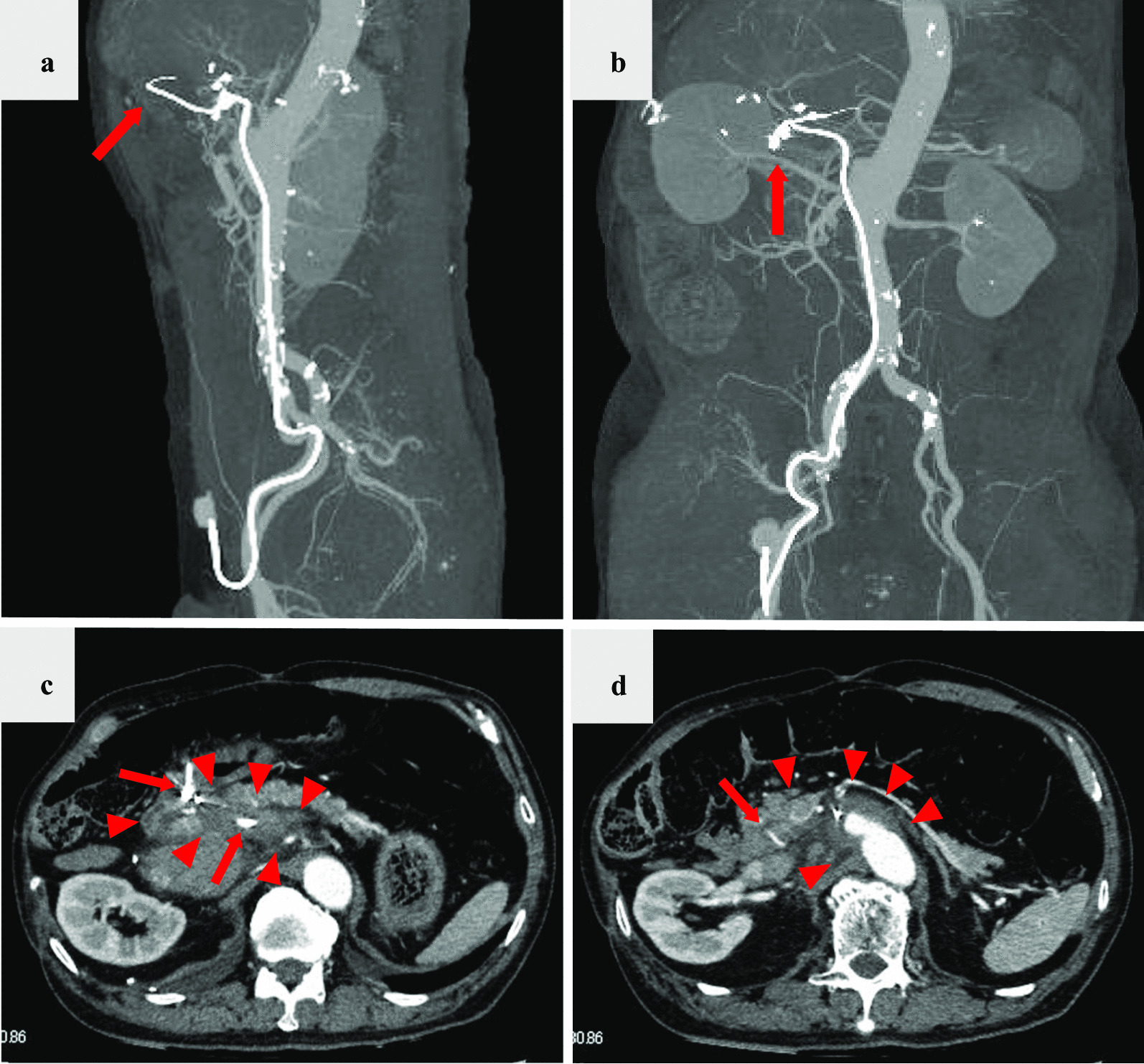
Computed tomography of the chest and abdomen reveals the thoracoabdominal aortic aneurysm along with the implanted arterial catheter inserted from the left femoral artery to the hepatic artery (**a**, **b** arrow). An irregularly shaped aortic aneurysm was identified at the origin of the celiac artery, with partially expanded common hepatic artery with disproportionate fat stranding (**c**, **d** arrowhead) along the catheter (**c**, **d** arrow); no extravasation was observed using contrast enhancement (**c**, **d**)

**Fig. 2:**
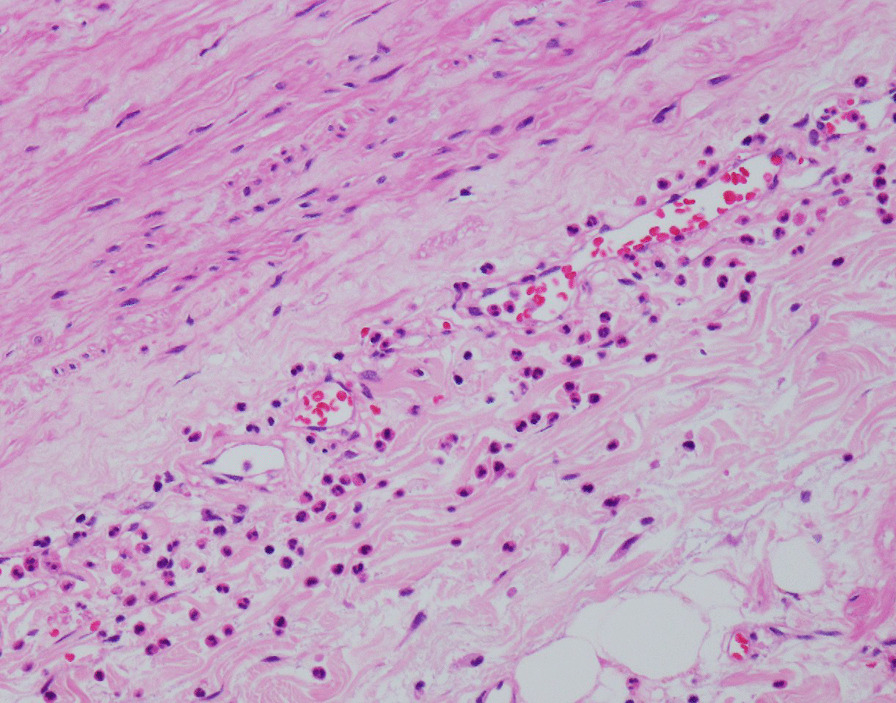
Histopathology examination of the aortic aneurysm wall confirmed an infected aortic aneurysm based on infiltration of neutrophils mainly in the small blood vessels around the adventitia and infiltration of neutrophils, lymphocytes, and plasma cells in the media of the blood vessels (hematoxylin and eosin staining)

On the day of admission, his blood pressure was 171/75 mmHg, heart rate was 67 bpm, SpO_2_ 97% at ambient room air, and body temperature was 36.6 °C. He denied abdominal pain, and pain or numbness in the lower extremities. General appearance was not in acute distress. There was no conjunctiva pallor or icterus. Respiratory sounds were clear to auscultation bilaterally and there were no wheezes or crackles. Cardiovascular examination revealed normal S1 and S2. There was no S3, S4, or murmurs. Abdominal examination revealed a flat and soft abdomen with audible bowel sounds. There was no bruit. There was no abdominal tenderness or hepatosplenomegaly. There was no spinal tenderness or costovertebral angle tenderness on percussion. There was no edema of his lower extremities. There was no joint swelling bilaterally at the wrists, ankles, and knees. General physical examinations revealed no abnormalities. His neurologic examination 2 to 12 were intact. There were no abnormalities with sensation and strength throughout with normal reflexes. Although laboratory analysis revealed normal results for complete blood count, electrolyte level, creatinine level, liver function, and coagulation test, levels of beta-d-glucan were slightly elevated at 24 pg/mL (reference value, < 20 pg/mL) (Table 1). Urinalysis was negative for proteinuria, pyuria, and hematuria (Table 1). Blood culture of aerobic and anaerobic bacteria including fungi and urine culture were all negative (Table 1). Transthoracic echocardiography revealed no valve vegetation, no valve regurgitation, no stenosis, and a normal ejection fraction. Computed tomography (CT) of the chest and abdomen revealed an irregularly shaped aortic aneurysm measuring 45 × 33 mm at the origin of the celiac artery and a partially expanded common hepatic artery with disproportionate fat stranding; no extravasation was observed using contrast enhancement (Fig. 1). There was a high possibility that the aortic aneurysm was infected because it was at the site of the catheter that was inserted for the femoral artery via the common hepatic artery. The patient was diagnosed with impending rupture of acute thoracoabdominal aortic aneurysm and was admitted to the intensive care unit of our hospital. Graft replacement was performed for the thoracoabdominal aortic aneurysm, and the implanted catheter was removed during surgery and tested for culture. Pus was discharged from the aortic aneurysm wall incision and collected with swab for culture. The cultures of both the removed catheter and the pus of the aneurysm revealed *Escherichia coli, Serratia marcescens*, *Eikenella corrodens*,* Streptococcus anginosus*, *α-Streptococcus*, and *Candida glabrata*. The reported antimicrobial sensitivities of these organisms are shown in Table 2. Antimicrobial susceptibilities were determined by the disk diffusion method, and the results were interpreted according to the Clinical and Laboratory Standards Institute (CLSI) guidelines. Results of pathology examination of the wall tissue of the aneurysm were compatible with those of the infected aneurysm cultures because the former showed infiltration of neutrophils mainly in the small blood vessels around the adventitia and infiltration of neutrophils, lymphocytes, and plasma cells in the media of the blood vessels (Fig. 2). On the basis of these findings, a diagnosis of catheter-related thoracoabdominal infected aortic aneurysm was made.

**Table 1:** Results of laboratory findings

Complete blood count			Biochemistry test			Urinalysis		
White blood cell	7.6	10^3^/μL	Total protein	7.8	g/dL	Dipstick		
Neutrophils	69.1	%	Albumin	3.6	g/dL	Color	Yellow	
Lymphocytes	22.0	%	Aspartate aminotransferase	16.0	IU/L	Specific gravity	1.024	
Eosinophils	0.8	%	Alanine aminotransaminase	12.0	IU/L	pH	5.5	
Basophils	0.8	%	Total bilirubin	0.4	mg/dL	Glucose	Negative	
Monocytes	7.3	%	Gamma-glutamyl transferase	20.0	IU/L	Protein	Negative	
Hemoglobin	12.3	g/dL	Alkaline phosphatase	341.0	IU/L	Bilirubin	Negative	
Hematocrit	39.1	%	Lactate dehydrogenase	216.0	IU/L	Ketones	+	
Platelets	40.5	10^4^/μL	Urea nitrogen	12.4	mg/dL	Hemoglobin	Negative	
			Creatinine	0.6	mg/dL	Nitrate	Negative	
Coagulation test			Sodium	134.0	mEq/L	Leukocyte esterase	+	
Prothrombin time	85.7	%	Potassium	4.3	mEq/L	Microscopy exam		
International normalized ratio	1.1		Chloride	93.0	mEq/L	Red blood cells	1–4	/HPF
d-Dimer	1.5	μg/mL	Calcium	9.3	mg/dL	White blood cells	1–4	/HPF
Fibrinogen	452.0	mg/dL	Phosphate	3.1	mg/dL	Epithelial cells	1–4	/HPF
Fibrin degradation products	3.9	μg/mL	Creatine kinase	92.0	IU/L	Casts	1–4	/HPF
			C-reactive protein	1.6	mg/dL	Crystals	Negative	
			Procalcitonin	0.1	ng/mL			
			Beta-d-glucan	24.0	pg/mL			
			Interferon-gamma release assays	Negative		Cultures		
			Triglyceride	91.0	mg/dL	Blood of aerobic	Negative	
			Total cholesterol	108.0	mg/dL	Blood of anaerobic	Negative	
			LDL-cholesterol	54.0	mg/dL	Urine	Negative	
			HDL-cholesterol	41.0	mg/dL	Removed implanted catheter	*	
			HbA1c	5.9	%	Aneurysm pus	*	
			HBs antigen	Negative				
			HCV antibody	Negative				
			HIV antigen/antibody	Negative				

*Refer to Table 2

**Table 2:** Result of antimicrobial susceptibility for causative pathogens from implanted catheter and aneurysm pus

Antimicrobial agent	*E. coli*		*S. marcescens*		*S. anginosus*		*α-Streptococcus*		*C. glabrata*	
	MIC (μg/mL)	Categorization	MIC (μg/mL)	Categorization	MIC (μg/mL)	Categorization	MIC (μg/mL)	Categorization	MIC (μg/mL)	Categorization
Benzylpenicillin					≤ 0.06	S	≤ 0.06	S		
Ampicillin	≤ 2	S	8	R	≤ 0.25	S	≤ 0.25	S		
Piperacillin	≤ 4	S	≤ 4	S						
Amoxicillin/clavulanate	≤ 2	S	4	R						
Ampicillin/sulbactam		S		R						
Piperacillin/tazobactam		S		S						
Cefazolin	≤ 4		≤ 4	R						
Cefaclor		S		R						
Cefmetazole	≤ 1	S	2	S						
Cefotiam	≤ 8	S	≤ 8	S						
Cefotaxime	≤ 1	S	≤ 1	S	≤ 0.12	S	≤ 0.12	S		
Ceftriaxone					0.5	S	≤ 0.06	S		
Ceftazidime	≤ 1	S	≤ 1	S						
Cefepime	≤ 1	S	≤ 1	S						
Cefditoren pivoxil		S								
Cefpodoxime proxetil	≤ 0.25	S	1	S						
Imipenem/cilastatin	≤ 0.25	S	≤ 0.25	S						
Meropenem	≤ 0.25	S	≤ 0.25	S						
Doripenem		S		S						
Gentamicin	≤ 1	S	≤ 1	S						
Amikacin	≤ 2	S	≤ 2	S						
Minocycline	≤ 1	S	2	S						
Tetracycline					0.5	S	0.5	S		
Erythromycin					≤ 0.12	S	≤ 0.12	S		
Fosfomycin	≤16	S	≤ 16	S						
Sulfamethoxazole/trimethoprim	≤ 20	S	≤ 20	S						
Clindamycin					≤ 0.25	S	≤ 0.25	S		
Levofloxacin			≤ 0.12	S	1	S				
Ciprofloxacin	≤ 0.25	S	≤ 0.25	S						
Vancomycin					0.5	S	0.5	S		
Linezolid					≤ 2	S	≤ 2	S		
Fluconazole	≤ 0.12	S	≤ 0.12	S	0.5	S			8	S
Amphotericin B									≤ 0.25	S
Flucytosine									≤ 1	S
Voriconazole									0.25	S
Micafungin									≤ 0.06	S
Caspofungin									≤ 0.25	S

*Eikenella corrodes* were not used for this antimicrobial susceptibility test

*MIC* minimum inhibitory concentration,* S *susceptible,* R* resistant

On the day of admission, antibiotics therapy was considered for implanted catheter-related infection. The patient was administered a combination of vancomycin 1.0 g intravenously every 12 hours, piperacillin/tazobactam 4.5 g every 6 hours, and micafungin 150 mg every 24 hours until all culture results were confirmed for a week at first. After determination of the drug susceptibilities of all strains, these antibiotics were found to be suitable and were continued. Although the thoracoabdominal aneurysm was resected and pus was drained, antibiotics were administered for 6 weeks in consideration of infection of the perivascular area from the celiac artery to the hepatic artery. His symptoms and laboratory test results improved after surgery and administration of antibiotics. The patient was discharged on day 45, and no recurrence of infected aortic aneurysm was observed on subsequent follow-up CTs as an outpatient for 1 year.

## Discussion

Although there are some reports of infections and pseudoaneurysms due to catheters [4], to the best of our knowledge, no study has reported infected thoracoabdominal aneurysms that are associated with implanted long-term arterial catheters for chemotherapy. This case was a rare but extreme scenario caused by a long-term indwelling aortic catheter compounded by rare organisms responsible for the infection.

The most prevalent microorganisms present in the arterial wall that are likely to cause infection of an aneurysm are *Staphylococcus* spp. and *Salmonella* spp. [5]. Other microorganisms include *Streptococcus pneumoniae*, *Treponema pallidum*, and *Mycobacterium tuberculosis*, as well as other bacterial, fungal, and anaerobic pathogens [6]. Infected aneurysms can occur in any artery but are most observed in the extremities, splanchnic, and cerebral circulations, often at the points of vessel bifurcation [7]. In the present case, cultures of the aortic aneurysm wall tissue revealed the causative pathogens as *E. coli, S. marcescens*, *E. corrodens, S. anginosus*, *α-Streptococcus,* and *C. glabrata*. There were several possible routes of infection related to implanted long-term arterial catheters, and the more likely ones were through the transhepatic artery, transhepatic portal vein, and transdermal infections, including bloodstream infections. Although cultures from the removed catheter and aneurysm pus revealed several pathogens, transient bacteremia associated with an implanted arterial catheter could have been controlled by autoimmunity. In addition, multiple pathogens may exist in the aortic arterial wall and may have caused the infected aortic aneurysm. Interestingly, it is considered that the celiac artery had a damaged arterial wall during insertion of the catheter, which eventually developed into an aneurysm, with inflammation and microorganisms spreading to the thoracoabdominal aorta. This was an extremely rare occurrence, and an infectious aortic aneurysm was suspected from the distortion of the aneurysm shape.

Several reports have investigated the risk factors for an infected aneurysm, and these include arterial injury, trauma, antecedent infection, endocarditis, preexisting aneurysm, impaired immunity, and advanced age [8, 9]. The patient’s past medical history and clinical examination did not indicate the presence of these risk factors. Either a venous or arterial indwelling catheter has an obviously high risk of catheter-related infection. In general, the diagnosis of an infected aneurysm is based upon imaging the aneurysm, and infection is confirmed by culturing an organism from the blood. CT angiography definitively diagnoses the aneurysm, specific features suggest infection, and CT also simultaneously evaluates the status of the circulation [10]. In this case, we considered that the thoracoabdominal aortic aneurysm was highly likely to be related to the implanted catheter on the basis of findings from CT imaging, the catheter and pus from aortic wall tissue cultures, and pathology examinations.

The standard treatment of most infected aneurysms is antibiotic therapy combined with surgical debridement with or without revascularization [11]. The initial choice of antibiotic therapy should be guided by the most likely infecting organism on the basis of the clinical circumstances. Antibiotics should be tailored to culture and susceptibility results when they become available. If surgical drainage is performed, this time period commences from the day of surgery. However, there are no data to support a specific duration of antibiotic therapy. In this case, we administrated a combination of antibiotics for 6 weeks on the basis of physical examination, laboratory findings, and follow-up CT findings.

In this era, because the number of patients has been increasing with catheter-based examination and treatment options, the number of patients with indwelling catheter infections is also expected to increase. Immediate confirmatory diagnosis and appropriate treatment are essential. As a matter of course, it is important to remove the catheter as soon as possible at the end of procedure to avoid this critical illness.

## Conclusion

This case was a rare but extreme scenario caused by a long-term indwelling catheter compounded by rare organisms responsible for the infection. Clinicians should be aware that long-term implanted arterial catheters can cause not only catheter-related bloodstream infections but may also be a risk factor for infected aortic aneurysms in rare cases.

## Data Availability

Not applicable.
